# Understanding the Molecular Epidemiology of Community-Acquired Methicillin-Resistant *Staphylococcus aureus* in Northern Saudi Arabia: A Spotlight on *SCCmec* and *spa* Typing

**DOI:** 10.1155/cjid/2753992

**Published:** 2025-05-24

**Authors:** Mishaal Alanazi, Hussein Aldughmani, Farhan Alrowaili, Fatma Alenazi, Marzwq Alrewaili, Mezna Alenazi, Abdulhamid Alsalmi, Hala M. Rushdy, Khalid Almaary

**Affiliations:** ^1^Department of Laboratories and Blood Bank, Gurayat Health Affairs, P.O. Box 965, Gurayat 75911, Saudi Arabia; ^2^Department of Central Sterile Services, Gurayat Dental Centre, P.O. Box 965, Gurayat 75911, Saudi Arabia; ^3^General Directorate of Infection Prevention & Control, Ministry of Health, Riyadh 12234, Saudi Arabia; ^4^Department of Botany & Microbiology, College of Science, King Saud University, P.O. Box 2460, Riyadh 11451, Saudi Arabia

## Abstract

**Background:** Methicillin-resistant *Staphylococcus aureus* (MRSA) is a strain resistant to certain antibiotics, making it difficult to treat. MRSA infections can occur in healthcare settings and in the wider community. The prevalence of MRSA strains has significantly increased in Saudi Arabia over the last 2 decades.

**Objective:** This study investigated the molecular epidemiology of MRSA isolates from patients at Gurayat General Hospital in Northern Saudi Arabia.

**Methods:** Ninety-seven MRSA isolates were collected from patients in 2018-2019. MRSA isolates were subjected to antibiotic susceptibility testing, SCC*mec* typing, *spa* typing, and pvl gene detection.

**Results:** All strains were susceptible to vancomycin, teicoplanin, and linezolid. Resistance to clindamycin (33%) and erythromycin (44%) was common. Resistance to ciprofloxacin (19%), gentamicin (14%), and tetracycline (19%) was also observed. Forty-four *spa* types were identified, with t304 (11.4%), t044 (8.4%), and t0127 (8.4%) being the most common. SCC*mec* types IVd (39%), IVc (27%), and V (24%) were most frequent. Additionally, the pvl gene was detected in 49% of the isolates.

**Conclusion:** Community-acquired MRSA clones are prevalent in the healthcare setting. The predominant genotype was t304 (10%), followed by t044 (7%) and t0127 (7%). The SCC*mec* IVd type was the most common type frequently associated with *spa* type t304, whereas SCC*mec* type IVc was primarily associated with *spa* type t044. Isolates harboring SCC*mec* type IVd showed more nonsusceptible phenotypes to ciprofloxacin than other SCC*mec* types. Most MRSA isolates were resistant to erythromycin and clindamycin. These findings provide valuable insights into the molecular epidemiology of MRSA in the northern region of Saudi Arabia and highlight the prevalence of specific MRSA strains and their antibiotic resistance profiles. This information is essential for monitoring and addressing the spread of MRSA in healthcare settings.

## 1. Introduction

Methicillin-resistant *Staphylococcus aureus* (MRSA) is a significant cause of community-acquired and hospital-acquired infections [1]. *Staphylococcus aureus* (*S. aureus*) is a considerable pathogen due to its virulence, invasiveness, and antibiotic resistance [2]. These characteristics are linked to virulence factors such as staphylococcal enterotoxins, hemolysins, coagulase, toxic shock syndrome toxin-1(TSS-1), exfoliative toxins, staphylococcal protein A (*spa*), and Panton–Valentine leucocidin (PVL) [3]. Colonization with *S*. *aureus* increases the risk of subsequent infection [4]. MRSA can cause a variety of infections, ranging from skin and soft tissue infections and food poisoning to severe conditions such as osteomyelitis, endocarditis, and toxic shock syndrome (TSS) [5].

Since its emergence in 1960, MRSA has spread globally, causing systemic infections in both hospital-associated MRSA (HA-MRSA) and the community-associated MRSA (CA-MRSA) [6]. Methicillin resistance is mediated by *mecA, mecI*, and *mecR* genes located on bacterial chromosomes within mobile genetic elements known as staphylococcal cassette chromosome mec (SSC*mec*) [3]. The *mec*A gene encodes the low-affinity penicillin-binding protein 2a (PBP2a), conferring resistance to all β-lactam antibiotics [1].

MRSA prevalence in Saudi Arabia has been notable over the last 2 decades [7]. Epidemiological surveys indicate that approximately 36% of *S. aureus* strains are MRSA, with CA-MRSA accounting for an estimated 23.2% of *S. aureus* carriage [8, 9]. In the northern region, MRSA accounts for 56.7% of *S. aureus* isolates [10]. HA-MRSA clones such as the Vienna/Hungarian/Brazilian clone (CC8/ST239-III) are predominant in Riyadh and Dammam [11]. CA-MRSA clones such as Barnim/UK-EMRSA-15 (CC22-IV), Southwest Pacific clone (ST30-IV), and European CA-MRSA clone (CC80-IV) have also been identified in Saudi Arabia and neighboring Gulf states [11–14].

While numerous molecular studies have been conducted in Saudi Arabia, research in the northern region remains limited. This study uses SCC*mec* and *spa* typing, a rapid and highly discriminatory technique [15], to identify common MRSA strains among hospitalized patients at the Gurayat General Hospital (GGH) in Northern Saudi Arabia.

## 2. Methods

### 2.1. Study Design and Sample Collection

Gurayat is the second-largest city in the northern Al-Jouf region of Saudi Arabia, near the Saudi border with Jordan. GGH is the only referral hospital in the city, serving a population of over 300,000, including neighboring villages. GGH has a capacity of 200 beds. This study was conducted at the Gurayat Regional Laboratory, a reference laboratory for the Al-Jouf region.

The study population included patients admitted to GGH, regardless of age or gender, who underwent surgery during the study period and subsequently developed signs and symptoms of surgical site infections. Outpatients with infected wounds and those receiving treatment were excluded from this study.

### 2.2. Sample Size Calculation

The sample size for the current study was calculated using Kish's formula for cross-sectional studies, based on a MRSA prevalence of 15% at GGH. A minimum sample size of 90 specimens was determined to be necessary. Between 2018 and 2019, nonduplicated MRSA isolates were collected from the study population.

### 2.3. Bacterial Isolates

Ninety-seven MRSA isolates associated with clinical infection were collected during 2018-2019 at GGH, Saudi Arabia. Isolates were identified as MRSA using VITEK 2 (*bioMérieux*, France). Susceptibility to oxacillin and cefoxitin was confirmed using oxacillin 1 μg disk and cefoxitin 30 μg disk according to the performance standards of the Clinical and Laboratory Standards Institute (CLSI). Clinical and demographic data were collected from patients' medical records. CA-MRSA was defined as an MRSA isolate obtained either from an outpatient or an inpatient within 48 h of hospitalization and without the patient having a medical history of MRSA infection or colonization. The isolates were detected from different clinical sources, including skin and soft tissue (cutaneous abscess and wound secretion), respiratory tract (sputum), blood, and ear.

Ethical approval was obtained from the hospital ethics committee (Research Project No. 064).

### 2.4. Antimicrobial Susceptibility Testing

The antimicrobial susceptibility was performed using the *bioMérieux* VITEK 2 system following the manufacturer's instructions. Results were interpreted following CLSI guidelines (CLSI, 2019). The following 12 drugs were tested: linezolid (LZD), ciprofloxacin (CIP), clindamycin (C), erythromycin (E), trimethoprim-sulfamethoxazole (SXT), moxifloxacin (MOF), vancomycin (V), teicoplanin (TEIC), tetracycline (TET), rifampicin (RF), gentamicin (GM), and daptomycin (DA). *S. aureus* ATCC29213 was used as a quality control.

### 2.5. SCC*mec* Typing

SCC*mec* typing was performed as previously described [16]. This was based on the asset of multiplex PCR primers to identify SCC*mec* genotypes belonging to I–III, IVa–IVd, IVh, and V. The protocol includes the detection of the methicillin-resistant *mec*A gene. MRSA isolates that could not be assigned to any expected type were defined as nontypable (NT). Primers for SCC*mec* typing are presented in Table S1 in the supporting information.

### 2.6. Detection of *pvl* Gene

The *pvl* gene was detected in MRSA isolates using previously described primers and protocols [17]. Primers for the *pvl* gene are presented in Table S1 in the supporting information.

### 2.7. *spa* Typing

The polymorphic X region of the *spa* gene was amplified and sequenced as described previously using the primers spA-1113f and spA-1514r [15]. Primers for *spa* typing are presented in Table S1 in the supporting information. The PCR products were purified using the QIAquick PCR Purification Kit (QIAGEN). The purified DNA product was then sequenced in both forward and reverse directions using the same PCR primers in combination with the 3500 XL Genetic Analyzer by the manufacturer's instructions (Applied Biosystems). The sequences obtained were analyzed and aligned using the Bio Edit program (Version 7.2). The *spa* typing was assigned by submitting the data to the *spa* databases (https://spatyper.fortinbras.us/) and (https://spaserver.ridom.de).

### 2.8. Statistical Analysis

Statistical analyses were performed using the Chi-square or Fisher's exact tests as appropriate for categorical data analysis. Statistical significance was set at *p* ≤ 0.05.

## 3. Results

### 3.1. MRSA Isolation and Antimicrobial Susceptibility Test

**Figure 1:**
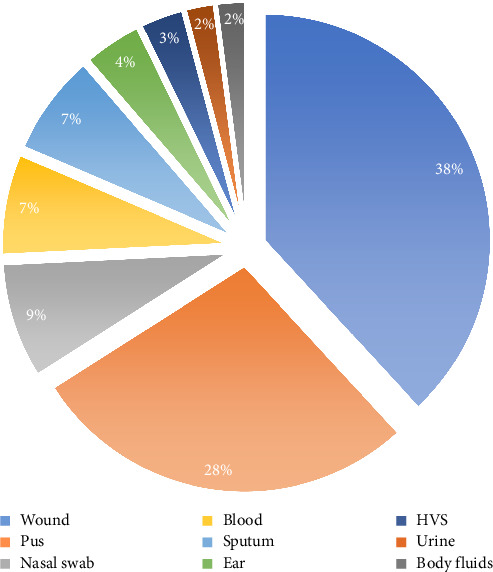
Distribution of MRSA according to isolation sites.

A total of 97 MRSA isolates were obtained from various clinical specimens, with the distribution by sample type as follows: wound (38%), pus (28%), nasal (9%), sputum (7%), blood (7%), and ear samples (4%). Isolates from urine, high vaginal swabs, and body fluids each represented less than 3% of the total (Figure 1). Among the isolates, 43% were obtained from female and 57% from male patients, indicating a slight male predominance across different wards of GGH.

All MRSA strains exhibited susceptibility to V, TEIC, LZD, RF, and DA. However, resistance was observed in 33% of isolates to C and 44% to E (Table 1). Other notable resistance rates included 19% to CIP, 7% to MOF, 14% to GM, 8% to SXT, and 19% to TET. The resistance to E and C was consistently high among all strains; in particular, SCC*mec* types IVc and IVd exhibited elevated TET resistance, with 67% (12/18) of these isolates demonstrating this phenotype. Notably, 56% (10/18) of CIP-resistant strains were associated with SCC*mec* type IVd. Interestingly, SXT resistance was exclusively found in SCC*mec* type V and NT strains, constituting 8% (8/97) of the total isolates (*p*=0.02; Table 2). Resistance to three or more antibiotics was observed in 16% and 26% of the type IVd and V strains, respectively.

### 3.2. Distribution of *spa* and SCC*mec* Types

**Figure 2:**
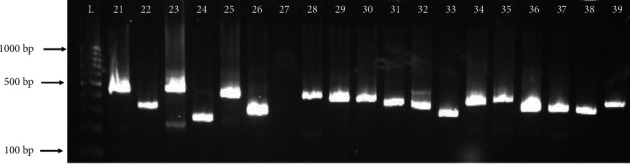
A representative gel demonstrating the *spa* PCR amplification typing variability in the band size, indicating variability in the number of repeats and genotypes. Lane L, 100-bp DNA ladder; Lanes 21–39: the variable PCR product of *spa* gene among MRSA isolates. Supporting File 2 presents other figures of *spa* typing among MRSA isolates.

Molecular characterization confirmed that all isolates were positive for the *mec*A gene. Out of 97 strains, 88 (91.2%) showed the *spa* gene PCR products with different sizes (Figure 2). The *spa* genotyping revealed substantial diversity among the MRSA strains, identifying 44 distinct *spa* types (Table 3). The most prevalent type was t304 (10 isolates, 11.4%), followed by t044 (seven isolates, 8.4%), t127 (seven isolates, 8.4%), t021 (five isolates, 5.7%), t131 (five isolates, 5.7%), t2319 (five isolates, 5.7%), and t002 (four isolates, 4.5%). All other 37 *spa* types were less frequent (≤ 3.0%), t311, t2297, t309, t4019, t458, t690, t042, t045, t084, t10234, t10892, t1109, t1234, t12659, t1277, t1379, t16606, t186, t1921, t19677, t203, t267, t315, t355, t376, t3841, t4198, t434, t5168, t7275, t9867, t748, t535, t903, t991, t504, and t008. A total of nine isolates could not be assigned to any *spa* type.

**Figure 3:**
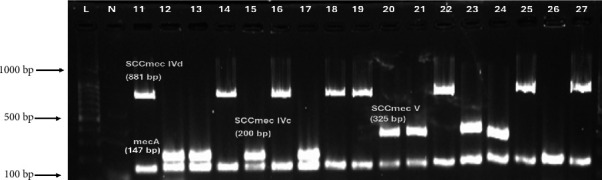
A representative gel demonstrating the multiplex PCR SCC*mec* typing of MRSA isolates (Lanes 11–27); Lane L, 100-bp DNA ladder; Lane N: negative control; the isolates that tested positive for the *mec A* gene are indicated by an amplicon of (147-bp); Lanes 11, 14, 16, 18, 19, 22, 25, and 27 represent SCC*mec* IVd amplicon (881-bp); Lanes 12, 13, 15, and 17 represent SCC*mec* IVc type amplicon (200-bp); Lanes 20, 21, 23, and 24 represent SCC*mec* V type amplicon (325-bp); Lane 26: no PCR product. Supporting File 2 presents other multiplex PCR SCC*mec* typing figures among MRSA isolates.

Analysis of SCC*mec* types showed the following distribution: Type IVd was the most prevalent at 39% (38/97), followed by type IVc at 27% (26/97) and type V at 24% (23/97), with 10% (10/97) of strains being NT (Figure 3 and Table 4). Among the isolates derived from wound samples, a significant fraction of IVc strains was present, accounting for 58% (15/26) and showing diverse *spa* types, with t044 being the most frequent. Notably, 93% (14/15) of these IVc strains tested positive for the *pvl* gene. Similarly, 29% (11/38) of IVd strains originated from wound specimens, predominantly linked to *spa* type t034, and 43% (10/23) of type V strains were identified from wound sites, with t021 being the predominant *spa* type.

Further analysis revealed that 57% (4/7) of blood samples were SCC*mec* IVd strains, while 26% of total IVd strains (10/38) were recovered from pus, with 40% (4/10) associated with *spa* type t127. Additionally, 35% (8/23) of type V strains were isolated from pus. Notably, 75% (6/8) of nasal samples were significantly associated with IVd strains (*p*=0.03, Table 4). No type V strains were found in sputum specimens, while both IVd and IVc strains were detected at comparable rates of 50% (3/6).

### 3.3. Association of *spa* and SCC*mec* Types With Infection Sites

Analysis of the association of *spa* and SCC*mec* type with infection sites is shown in Table 4. No significant associations were identified between *spa* types and infection sites. However, source analysis significantly showed that 58% (15/26) of IVc isolates were obtained from the wound site (*p*=0.02; Table 4), mainly linked to spa types t044, 27% (4/15). Moreover, specific *spa* types, such as t304 and t044, were found to be exclusively associated with SCC*mec* types IVd and IVc, respectively.

### 3.4. Association of SCC*mec* Type and *pvl*-Positive Strains With Infection Sites

**Figure 4:**
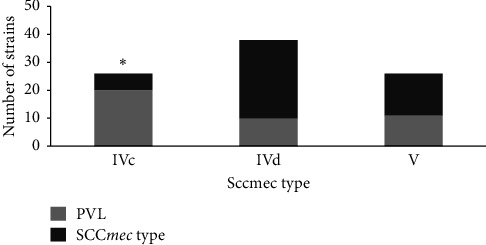
Prevalence of MRSA SCC*mec* types and *pvl* detection gene at the Gurayat General Hospital over 2 years (2018 and 2019). Notes: ^∗^*p* < 0.05.

In this study, the positivity for the *pvl* gene was found in 49% (48/97) of the total MRSA isolates (Table 5). There was a higher prevalence of 48% (23/48) among isolates from wound samples, with this detection rate being significantly associated with a value of 0.04. Among the *pvl*-positive strains, 42% (20/48) belonged to SCC*mec* type IVc. The detection rate was exceptionally high in wound specimens, reaching 70% (14/20), with a value of 0.02 (Table 4). The *spa* type t044 accounted for 35% (7/20) of the type IVc strains identified from wound specimens. In contrast, the *spa* type t021 was frequently associated with *pvl*-positivity among type V strains. The prevalence of the *pvl* gene in IVc was significantly higher than that in other types (Figure 4).

## 4. Discussion

The epidemiology of MRSA, particularly its geographic diversity and evolving dominant clones, is a crucial study area. This investigation, conducted at GGH in Saudi Arabia, provides valuable insights into the local prevalence of specific *spa* types and SCC*mec* elements. It contributes to a broader understanding of MRSA's distribution globally.

CA-MRSA infections are identified when MRSA is isolated within the first 48–72 h of hospitalization. It is well-established that SCC*mec* Types I, II, and III are associated with HA-MRSA, while CA-MRSA strains typically carry Types IV or V [18]. The study revealed a notable presence of frequent MRSA clones at GGH, emphasizing the significance of molecular characterization in this region. The predominant clone identified was SCC*mec* IVd, typically associated with *spa* type t304. Additionally, SCC*mec* type IVc, linked to *spa* type t044, was also prevalent, indicating a significant presence of CA-MRSA strains within the hospital environment.

The incidence of MRSA in Saudi Arabia varies notably by region, with prevalence rates reported as 42% in the Western region, 32% in the Central region, and 27% in the Eastern region [19]. A significant increase in CA-MRSA rates was observed in the Eastern region, rising from 23% in 2006 to 60% in 2015 [20]. In the larger context of the Arabian Gulf, prevalence rates have been reported between 15% and 35% [21–23]. All MRSA isolates in our study were classified as CA-MRSA and exhibited susceptibility to LZD, TEIC, and V. However, resistance rates to C and E were alarmingly high at 33% and 44%, respectively. These figures are higher than those reported in Riyadh and similar to the findings in Taif [8, 24]. CA-MRSA strains are still distinguished from HA-MRSA strains as CA-MRSA usually has limited sensitivity to β-lactam rather than other classes of antimicrobials [25]. In this study, resistance to other tested antibiotics varied. Still, notable resistance was observed for CIP, SXT, and TET, which reflects trends noted in previous studies across Saudi Arabia and the Arabian Gulf [22, 23, 26]. Low susceptibility rates to C, E, and CIP may be attributed to their high prescription rates, suggesting that their use for empiric MRSA coverage may no longer be justified.

PVL has emerged as a significant virulence factor associated with CA-MRSA, highlighting its clinical importance in *S. aureus* infections [27]. The prevalence of MRSA carrying *pvl* in Saudi Arabia has been highlighted in several studies, indicating that it is significantly high in most CA-MRSA infections [14, 28]. This study found a concerning 49% prevalence of the *pvl* gene in CA-MRSA isolates. The gene was more common in SCC*mec* type IVc (42%) than in type IVd (20%), primarily associated with spa type t044. In Saudi Arabia, many cases of infections linked to *pvl*-positive CA-MRSA strains of the IV types have been documented in healthcare settings. These cases are primarily represented by lineages such as CC22-IV (UK-EMRSA-15/Barnim Epidemic strain), CC30-IV (Southwest Pacific clone), and CC80-IV (European CA-MRSA clone) [11, 29]. The western region reported that 29% of MRSA infections were of Type IV, with 47.3% testing positive for the *pvl* gene [30]. In contrast, the eastern region showed an even higher prevalence, with 77.3% of MRSA isolates belonging to SCC*mec* Type IV [31]. These findings emphasize the increasing incidence of CA-MRSA infections in healthcare contexts and validate previous research, highlighting the urgent need for comprehensive surveillance initiatives. Such efforts are essential for accurately assessing the prevalence of IV genotypes in hospitals and understanding their implications for infection control practices.

The geographic variability of specific *S. aureus* clones is evident in Saudi Arabia and neighboring countries [11, 13, 19, 22, 32–38], as summarized in Table 6. Distinct *spa* types and SCC*mec* elements dominate various countries and regions. Our study identified t304-IVd as the most predominant clone, followed by t044-IVc, t127-IVd, t021-V, t2319-IVc, and t002-IVc. Notably, the clone's *spa* types t304, t044, and t127 were most detected in the eastern region, while t044 was most prevalent in Riyadh of the central region [39, 40]. Comparing our findings with other studies, Strains t304 and t044 were similarly identified in neighboring countries, suggesting a regional adaptation or spread of these clones within the Arabian Gulf. For instance, 30% of MRSA isolates in Iraq were identified as t304, the leading strain, alongside Strains t307, t346, and t044 [38]. Additionally, a study in Oman highlighted that the *pvl*-negative ST6-IV/t304 clone was the most frequently isolated strain, whereas ST80-IV/t044 and ST30-IV/t021 were detected less often [41]. A study in Kuwait reported different *spa* types among 667 isolates; *spa* types t304, t127, t044, t311, and t002 were the most frequently detected clones [42]. These findings across various regions underscore the complex landscape of MRSA epidemiology and emphasize the necessity for continued monitoring and intervention strategies.

Type t304 and t044 clones were classified as CA-MRSA, representing ST6 and ST80 MRSA, respectively [43]. The t304 clone, prevalent in the Middle East, has emerged as a significant strain in Europe, often associated with Middle Eastern immigrants, primarily classified as CA-MRSA with SCC*mec* IV and typically *pvl*-negative [44]. While most t304 isolates in our study showed negative *pvl* gene and susceptibility to a wide array of antimicrobials, some exhibited resistance to E, indicating a potential area of concern. In contrast, the t044 clone, originating from the Middle East and North Africa, is commonly found in the Arabian Gulf and the nearby regions. In Lebanon, it is associated with SCC*mec* IVc and the *pvl* gene [45]. This clone is also frequently reported in neighboring countries such as Jordan [46, 47]. Interestingly, unlike the typical CC80/t044 MRSA-IV clone, our findings revealed that t044 isolates demonstrated a high susceptibility to TET, suggesting possible therapeutic options [48]. Meanwhile, the t127 clone remains a significant challenge in Europe due to its multidrug-resistant tendencies [49]. However, our study uniquely found that only two t127 clones were classified as multidrug resistant, highlighting the variability within MRSA clones and the critical need for ongoing surveillance and characterization to inform effective treatment strategies.

Globally, there is a growing trend of HA-MRSA displacement by CA-MRSA clones. This shift has prompted calls for enhanced monitoring, control, and genomic surveillance of MRSA strains within hospitals. Al Yousef and Taha reviewed the increasing identification of CA-MRSA clones in Riyadh regions, including Barnim/UKEMRSA-15, Southwest Pacific clone, and European CA-MRSA clone [11]. Similar studies in Kuwait and the United Arab Emirates have reported the replacement of HA-MRSA clone ST239-III by CA-MRSA clones [50, 51]. In Southeast Asia, ST239-III and ST241-III have been replaced by ST22-IV and *pvl*-positive ST30-IV [52]. These observations challenge the traditional definition of MRSA infections as CA-MRSA based solely on the time of admission to healthcare settings [53]. It suggests that healthcare-associated risk factors may be more relevant in classifying infections.

## 5. Conclusion

The findings from this study underscore the importance of ongoing surveillance and molecular characterization of *S. aureus*, particularly MRSA strains, within healthcare settings. The high prevalence of CA-MRSA strains, coupled with significant resistance rates to commonly used antibiotics, highlights the urgent need for revised treatment guidelines and effective infection control measures. Given the geographic variability in MRSA clones, the data suggest that regional public health strategies are essential for addressing the challenges posed by MRSA in Saudi Arabia and the broader Arabian Gulf region. As CA-MRSA continues to emerge as a significant concern, efforts to monitor its spread and adapt therapeutic approaches will be critical to reduce the burden of infections in both community and healthcare settings.

## Figures and Tables

**Table 1:** Susceptibility and resistance pattern of 97 MRSA isolates to 12 antimicrobial agents.

Antimicrobials	MIC range (μg/mL)	Resistant (%)	Sensitive (%)
Ciprofloxacin	≤ 1–4	19	81
Clindamycin	≤ 0.5–4	33	67
Erythromycin	≤ 0.5–8	44	56
Gentamicin	≤ 4–16	14	86
Daptomycin	≤ 1	0	100
Rifampin	≤ 1–4	0	100
Linezolid	≤ 4–8	0	100
Moxifloxacin	≤ 0.5–2	7	93
Tetracycline	≤ 4–16	19	81
Trimethoprim-sulfamethoxazole	≤ 2/38–4/76	8	92
Teicoplanin	≤ 8–32	0	100
Vancomycin	≤ 2–16	0	100

**Table 2:** Comparison of resistance pattern among MRSA SCC*mec* types.

Antimicrobials	IVc	IVd	V	Nontypable	*p* value^∗^
	(*n* = 26)	(*n* = 38)	(*n* = 23)	(*n* = 10)	
	(27%)	(39%)	(24%)	(10%)	
Ciprofloxacin	2	10	6	0	0.07
Clindamycin	12	11	7	2	0.41
Erythromycin	12	15	13	3	0.48
Gentamicin	2	3	6	3	0.07
Moxifloxacin	0	5	2	0	0.19
Tetracycline	7	5	3	3	0.33
Trime-sulf	0	0	6	2	0.02
MDR > 3^a^	1	6	6	1	0.15

^∗^Fisher's exact test.

^a^Multidrug resistant to more than three antimicrobial agents.

**Table 3:** *Spa* type distribution among MRSA SCC*mec* types according to clinical samples.

SCC*mec* types, *n* (%)	*spa* type	No. of strains									
		Wound	Pus	Nasal swab	Blood	Sputum	Ear	HVS	Urine	Body fluids	Total (%)
SCCmec IVc, 26 (27%)	t002	2	1	—	—	—	—	—	—	—	3 (11.5)
	t042	1	—	—	—	1	—	—	—	—	2 (7.7)
	t044	4	1	1	—	1	—	—	—	—	7 (26.9)
	t10892	—	1	—	—	—	—	—	—	—	1 (3.8)
	t131	2	—	—	—	—	—	—	—	—	2 (7.7)
	t203	1	—	—	—	—	—	—	—	—	1 (3.8)
	t2319	1	1	—	1	1	—	—	1	—	5 (19)
	t376	—	—	—	1		—	—	—	—	1 (3.8)
	t434	1	—	—	—	—	—	—	—	—	1 (3.8)
	t458	2	—	—	—	—	—	—	—	—	2 (7.7)
	Not assigned	1	—	—	—	—	—	—	—	—	1 (3.8)

SCCmec IVd, 38 (39%)	t002	1	—	—	—	—	—	—	—	—	1 (2.6)
	t045	—	—	—	1	—	—	—	—	—	1 (2.6)
	t10234	—	1	—	—	—	—	—	—	—	1 (2.6)
	t12659	—	1	—	—	—	—	—	—	—	1 (2.6)
	t127	1	4	1	—	—	—	—	—	—	6 (16)
	t131	1	1	—	—	—	—	—	—	—	2 (5)
	t186	—	—	—	1	—	—	—	—	—	1 (2.6)
	t19677	1	—	—		—	—	—	—	—	1 (2.6)
	t304	4	1	2	1	2	—	—	—	—	10 (26)
	t309	—	1	—	—	—	—	—	—	1	2 (5)
	t3841	—	—	—	—	—	—	1	—	—	1 (2.6)
	t5168	—	—	1	—	—	—	—	—	—	1 (2.6)
	t690	1	—	—	—	—	1	—	—	—	2 (5)
	t7275	—	—	—	1	—	—	—	—	—	1 (2.6)
	t9867	—	—	—	—	1	—	—	—	—	1 (2.6)
	Not assigned	2	1	2	—	—	1	—	—	—	6 (15.8)

SCCmec V, 23 (24%)	t008	—	1	—	—	—	—	—	—	—	1 (4.3)
	t021	4	1	—	—	—	—	—	—	—	5 (21.7)
	t084	—	1	—	—	—	—	—	—	—	1 (4.3)
	t1234	—	1	—	—	—	—	—	—	—	1 (4.3)
	t1277	—	—	1	—	—	—	—	—	—	1 (4.3)
	t16606	—	1	—	—	—	—	—	—	—	1 (4.3)
	t1921	—	—	—	—	—	—	—	1	—	1 (4.3)
	t2297	1	—	—	—	—	1	—	—	—	2 (8.7)
	t267	1	—	—	—	—	—	—	—	—	1 (4.3)
	t311	1	1	—	—	—	—	1	—	—	3 (13)
	t315	1	—	—	—	—	—	—	—	—	1 (4.3)
	t4019	1	—	—	—	—	—	—	—	—	1 (4.3)
	t4198	1	—	—	—	—	—	—	—	—	1 (4.3)
	t748	—	—	—	1	—	—	—	—	—	1 (4.3)
	Not assigned	—	2	—	—	—	—	—	—	—	2 (8.7)

Not typed, 10 (10%)	t131	—	1	—	—	—	—	—	—	—	1 (10)
	t1379	—	—	—	—	—	—	1	—	—	1 (10)
	t127	—	—	—	—	—	—	—	—	1	1 (10)
	t4019	—	1	—	—	—	—	—	—	—	1 (10)
	t1109	—	—	—	—	1	—	—	—	—	1 (10)
	t535	—	—	—	—	—	1	—	—	—	1 (10)
	t903	—	1	—	—	—	—	—	—	—	1 (10)
	t991	1	—	—	—	—	—	—	—	—	1 (10)
	t504	—	1	—	—	—	—	—	—	—	1 (10)
	t355	—	1	—	—	—	—	—	—	—	1 (10)

**Table 4:** Frequency of MRSA SCCmec types in different clinical samples.

SCC*mec* types	Specimen type, *n* (%)										
	Wound	Pus	Nasal swab	Blood	Sputum	Ear	HVS	Urine	Body fluids	Total, *n* (%)	*p* value
SCC*mec* IVc	15 (58)	4 (15)	1 (3.8)	2 (8)	3 (11.5)	0 (0)	0 (0)	1 (3.8)	0 (0)	26 (27)	0.02
SCC*mec* IVd	11 (29)	10 (26)	6 (16)	4 (10.5)	3 (8)	2 (5)	1 (2.6)	0 (0)	1 (2.6)	38 (39)	0.03
SCC*mec* V	10 (42)	8 (35)	1 (3)	1 (4.3)	0 (0)	1 (4)	1 (4.3)	1 (4.3)	0 (0)	23 (24)	> 0.05
Not typed	1 (10)	5 (50)	0 (0)	0 (0)	1 (10)	1 (10)	1 (10)	0 (0)	1 (10)	10 (10)	> 0.05
Total, (%)	37 (38)	27 (28)	8 (8)	7 (7)	7 (7)	4 (4)	3 (3)	2 (2)	2 (2)	97	

**Table 5:** Association of SCC*mec* type and *pvl-*positive strains concerning infection sites.

MRSA source	IVc		IVd		V		Nontypable		Total *n*, (%)	*p* value^∗^
	(*n* = 26)		(*n* = 38)		(*n* = 23)		(*n* = 10)			
	^a^pvl +	pvl −	pvl +	pvl −	pvl +	pvl −	pvl +	pvl −	pvl +	
Wound	14	1	2	9	7	3	0	1	23 (48%)	0.04
PUS	3	1	3	7	3	5	5	0	14 (29%)	0.7
Nasal swab	1	0	1	5	0	1	0	0	2 (4.1%)	0.14
Blood	1	1	2	2	0	1	0	0	3 (6.2%)	0.7
Sputum	1	2	0	3	0	0	1	0	2 (4.1%)	0.25
EAR	0	0	1	1	0	1	0	1	1 (2.1%)	0.31
HVS	0	0	0	1	0	1	0	1	0	0.081
Urine	0	1	0	0	1	0	0	0	1 (2.1%)	0.9
Body fluids	0	0	1	0	0	0	1	0	2 (4.1%)	0.15
Total	20	6	10	28	11	12	7	3	48/97 (49%)	

^∗^Chi-square test.

^a^pvl, Panton–valentine leucocidin gene.

**Table 6:** Diversity of *spa* types and SCC*mec* types in Saudi Arabia and other countries.

Country	Most prevalent *spa* type(s)	Most prevalent SCC*mec* type(s)	Reference(s)
Saudi Arabia	t304, t044, t127	IVd, IVc, V	This study
Saudi Arabia	t304, t044, t127, t002, t2319	III, IV, V	[11, 19]
Kuwait	t304, t127, t044, t311, t002	N/A	[36]
Qatar	t852, t044, t657, t7358, t690	IV, V	[14]
Oman	ST6-IV/t304	IV	[22]
Jordan	t223, t386	IVa, IVc	[37]
Lebanon	t021, t044, t267	IVc	[32]
Iraq	t304	N/A	[38]
Iran	t030, t021	III	[33]
Africa	t012, t037, t045, t1476	I, II, III, V, VI	[34]
China	t437, t034, t011,t1250	IV, V	[35]

*Note:* N/A indicates that the SCCmec type was not specified in the reference or that there was no single most prevalent type. The *spa* types listed are not exhaustive but represent the most common types reported in the studies. Some studies may have identified multiple *spa* types and SCC*mec* types within a single country, reflecting the diversity of MRSA strains.

## Data Availability

The article and supporting information include the data generated or analyzed during this study.

## Supporting Information

Supporting Information 1 Supporting File S1: A table of the primers used to amplify the SCC*mec*, *spa*, and PVL genes.

Supporting Information 2 Supporting File S2: Gel electrophoresis results for PCR analysis, showing the SCC*mec*, *spa*, and PVL PCR products from the MRSA isolates.
