# Supplementary material for: Understanding the Molecular Epidemiology of Community-Acquired Methicillin-Resistant Staphylococcus aureus in Northern Saudi Arabia: A Spotlight on SCCmec and spa Typing
Source: Can J Infect Dis Med Microbiol. 2025 May 24;2025:2753992. doi: 10.1155/cjid/2753992 (PMC12126273; doi:10.1155/cjid/2753992)
Supplement: Supporting Information 1 — Supporting File S1: A table of the primers used to amplify the SCCmec, spa, and PVL genes. [file 2753992.f1.pdf]

S1: List of primers for molecular Epidemiology of MRSA

Primers for SCCmec typing						Ref
No	Primers (µM):	Vol/ul	Oligonucleotide sequence	Amplicon size (bp)	Specificity	J. A. Mcclure-Warnier, J. M. Conly, and K. Zhang, “Multiplex PCR assay for typing of staphylococcal cassette Chromosome mec types I to V in Methicillin-resistant Staphylococcus aureus,” J. Vis. Exp., no. 79, 2013.
1	Type I-F	0.25	GCTTTAAAGAGTGTCGTTACAGG	613	SCCmec I	
2	Type I-R	0.25	GTTCTCTCATAGTATGACGTCC			
3	Type II-F	0.25	CGTTGAAGATGATGAAGCG	398	SCCmec II	
4	Type II-R	0.25	CGAAATCAATGGTTAATGGACC			
5	Type II-F2	0.2	TAGCTTATGGTGCTTATGCG	128	SCCmec II, VIII	
6	Type II-R2	0.2	GTGCATGATTTCATTTGTGGC			
7	Type III-F	0.33	CCATATTGTGTACGATGCG	280	Mercury element of SCCmec III	
8	Type III-R	0.33	CCTTAGTTGTCGTAACAGATCG			
9	Type III-F5	0.4	TTCTCATTGATGCTGAAGCC	257	SCCmec III and IIIA	
10	Type III-R6	0.4	GTGTAATTTCTTTTGAAAGATATGG			
11	Type IVa-F	0.25	GCCTTATTCGAAGAAACCG	776	SCCmec IVa	
12	Type IVa-R	0.25	CTACTCTTCTGAAAAGCGTCG			
13	Type IVb-F	0.7	TCTGGAATTACTTCAGCTGC	493	SCCmec IVb, IIA, IIB, IIC, IIE	
14	Type IVb-R	0.7	AAACAATATTGCTCTCCCTC			
15	Type IVc-F2	0.25	CCTGAATCTAAAGAGATACACCG	200	SCCmec IVc, IVE	
16	Type IVc-R2	0.25	GGTTATTTTCATAGTGAATCGC			
17	Type IVd-F5	1.8	CTCAAAATACGGACCCCAATACA	881	SCCmec IVd	
18	Type IVd-R6	1.8	TGCTCCAGTAATTGCTAAAG			
19	Type IVE-F3	0.75	CAGATTCATCATTTCAAAGGC	175	SCCmec IVE, IVF	
20	Type IVE-R4	0.75	AACAACTATTAGATAATTTCCG			
21	Type V-F	0.33	GAACATTGTTACTTAAATGAGCG	325	SCCmec V	
22	Type V-R	0.33	TGAAAGTTGTACCCTTGACACC			
23	ccr4-Fd	0.35	ATCGCTCATTATGGATACYGC	106	SCCmec IIA, IIB, IIC, IIE, IVE, IVF, VI, VIII	
24	ccr4-R5	0.35	CCATTTTTTGATAACCTGAACG			
25	ccr4-R6	0.35	CTATTTTTTTATAGCCTGAACG			
26	MecA147-F	0.6	GTGAAGATATACCAAGTGATT	147	mecA	
27	MecA147-R	0.6	ATGCGCTATAGATTGAAAGGAT			

Primers for Spa Typing		Ref
s spa-1113f	5'- TAA AGA CGA TCC TTC GGT GAG C -3'	D. Harmsen et al., “Typing of Methicillin-Resistant Staphylococcus aureus in a University Hospital Setting by Using Novel Software for spa Repeat Determination and Database Management,” J. Clin. Microbiol., vol. 41, no. 12, 2003.
spa-1514r	5'- CAG CAG TAG TGC CGT TTG CTT -3'	

pvl primers			Ref
Luk PV-F	ATCATTAGGTAAAATGTCTGGACATGATCCA	**Amplicon size (bp)**	G. Lina et al., “Involvement of Panton-Valentine leukocidin-producing Staphylococcus aureus in primary skin infections and pneumonia,” Clin. Infect. Dis., vol. 29, no. 5, 1999.
Luk PV-R	GCATCAAGTGTATTGGATAGCAAAAGC	433	
