# Supplementary material for: Understanding the Molecular Epidemiology of Community-Acquired Methicillin-Resistant Staphylococcus aureus in Northern Saudi Arabia: A Spotlight on SCCmec and spa Typing
Source: Can J Infect Dis Med Microbiol. 2025 May 24;2025:2753992. doi: 10.1155/cjid/2753992 (PMC12126273; doi:10.1155/cjid/2753992)
Supplement: Supporting Information 2 — Supporting File S2: Gel electrophoresis results for PCR analysis, showing the SCCmec, spa, and PVL PCR products from the MRSA isolates. [file 2753992.f2.pdf]

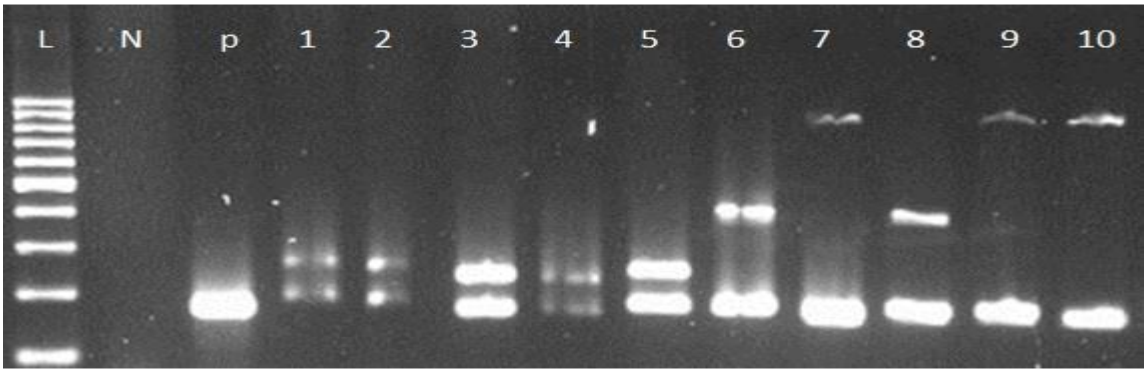

Figure 1: lane L, 100-bp DNA ladder ; lane N : negative control ; lane P: mec A positive control amplicon (147-bp) ; Lane 1,2,3,4 and 5 : the SCCmec IVc type amplicon (200-bp) ; lane 6 and 8: SCCmec V type amplicon (325-bp; lane 7,9 and 10: SCCmec IVd type amplicon (881-bp).

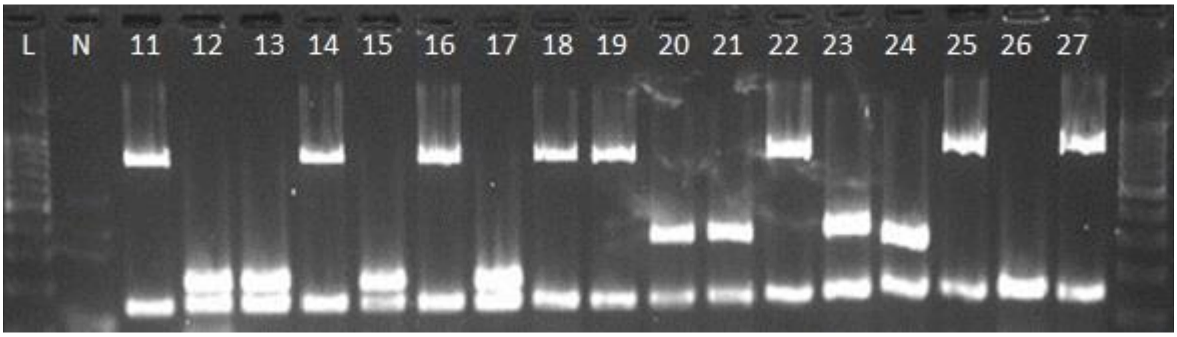

Figure 2: lane L, 100-bp DNA ladder ; lane N : negative control; mec A amplicon (147-bp); lane 11,14,16,18,19,22,25 and 27: the SCCmec IVd amplicon (881-bp); lane 12,13,15 and 17: SCCmec IVc type amplicon (200-bp); lane 20,21,23 and 24 : SCCmec V type amplicon (325-bp); lane 26: no PCR product.

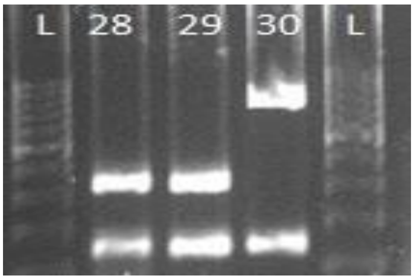

Figure 3: Lane L, 100-bp DNA ladder ; mec A amplicon (147-bp); lane 28 and 29 : SCCmec V type amplicon (325-bp); lane 30: SCCmec IVd type amplicon (881-pb).

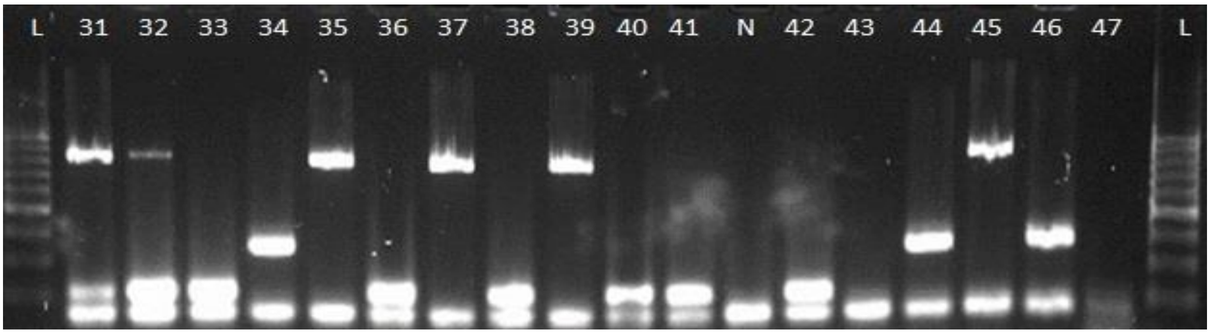

Figure 4:Lane L, 100-bp DNA ladder ; mec A amplicon (147-bp); lane 31, 35, 37, 39 and 45: the SCCmec IVd type amplicon (881-bp); lane 32, 33, 36, 38, 40, 41 and 42 : SCCmec IVc type amplicon (200-bp); lane 34, 44 and 46 : SCCmec V type amplicon (325-bp); lane N: negative control; lane; lane 43 and 47: no PCR product.

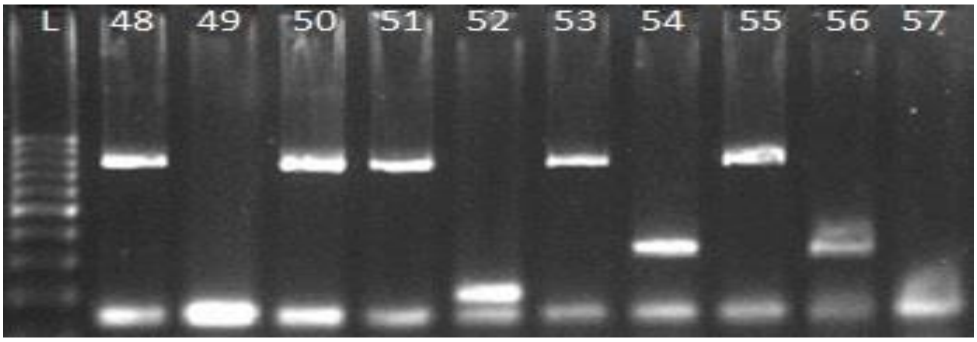

Figure 5: Lane L, 100-bp DNA ladder ; mec A amplicon (147-bp); lane 48,50,51,53 and 55: the SCCmec IVd type amplicon (881-bp) ; lane 52 : SCCmec IVc type amplicon (200-bp); lane 54 and 56: SCCmec V type amplicon (325-bp); lane 49 and 57: noPCR product.

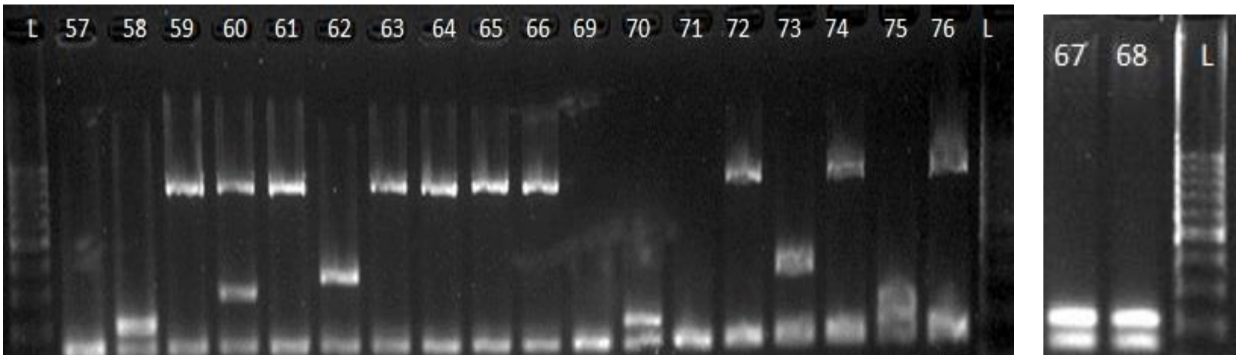

Figure 6: Lane L, 100-bp DNA ladder ; mec A amplicon (147-bp); lane 57,69 and 71; no PCR product; lane 58,67,68, 70 and 75: the SCCmec IVc type amplicon (200-bp); lane 59, 60, 61, 63, 64, 65,66,72,74 and 76 : SCCmec IVd type amplicon (881-bp); lane 62 and 73 : SCCmec V type amplicon (325-bp).

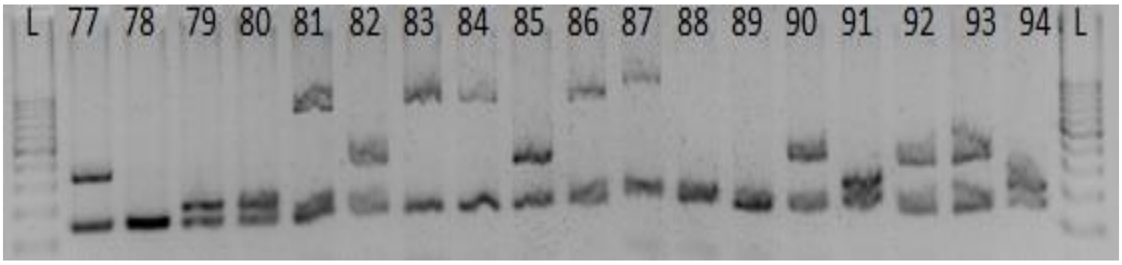

Figure 7: lane L, 100-bp DNA ladder mec A amplicon (147-bp); lane 77,82 ,85 ,90 ,92 and 93: the SCCmec IVd type amplicon (325-bp); lane 79 ,80 ,91 and 94 : SCCmec IVc type amplicon (200-bp); lane 81, 83, 84, 86 and 87 : SCCmec IVd type amplicon (881-bp); lane 78, 88 and 89: noPCR product.

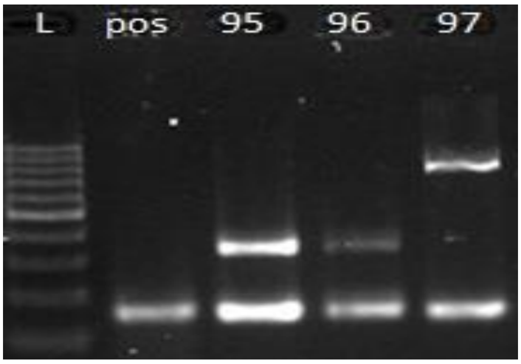

Figure 8: Lane L, 100-bp DNA ladder ; lane pos: mec A positive control amplicon (147-bp) ; lane 95 and 96: the SCCmec V type amplicon (325-bp); lane 97:the SCCmec V type amplicon (881-bp) .

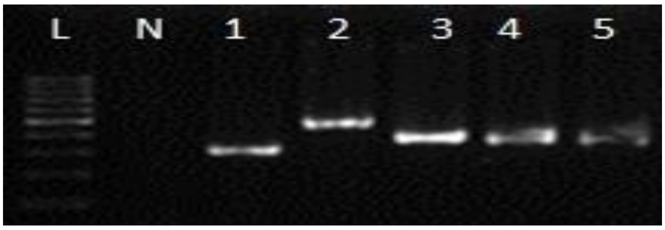

Figure 9: Lane L, 100-bp DNA ladder; lane N: negative control; lanes 1-5: the variable PCR product of spa.

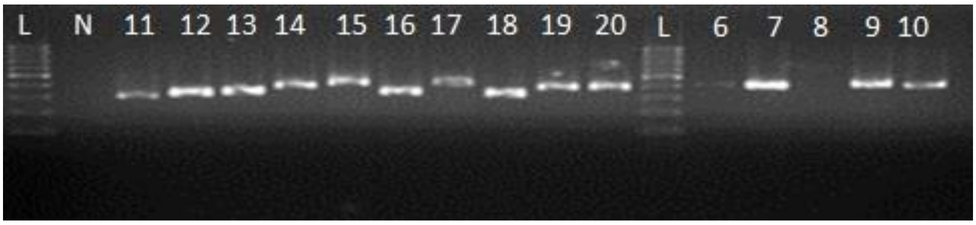

Figure 10: lane L, 100-bp DNA ladder; lane N: negative control; lanes 11-20; lanes 6-10: the variable PCR product of spa.

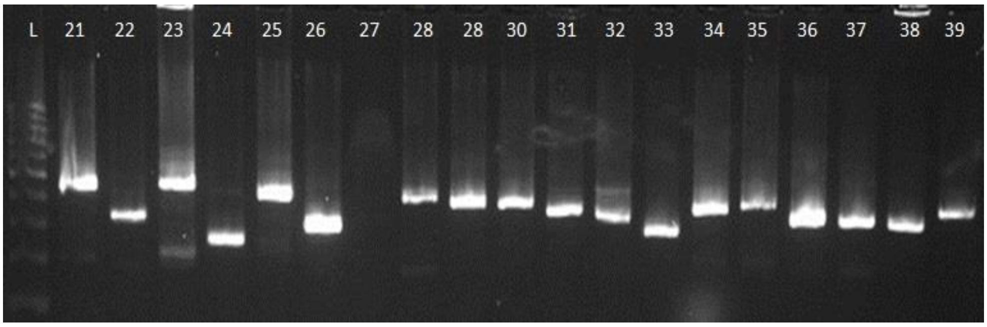

Figure 11: lane L, 100-bp DNA ladder; lanes 21-39: the variable PCR product of spa.

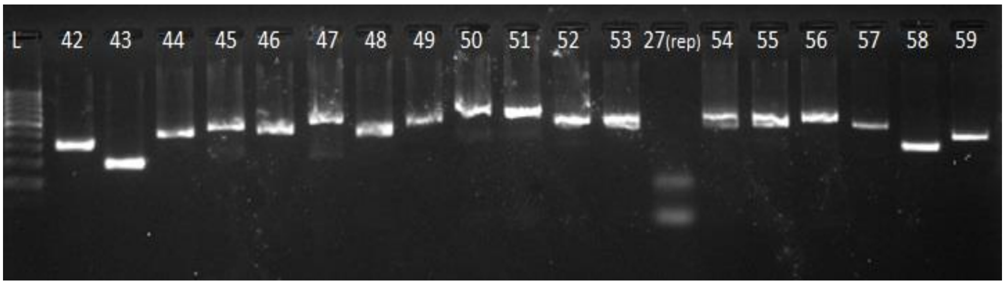

Figure 12. Lane L, 100-bp DNA ladder; lanes 42-59: the variable PCR product of spa.

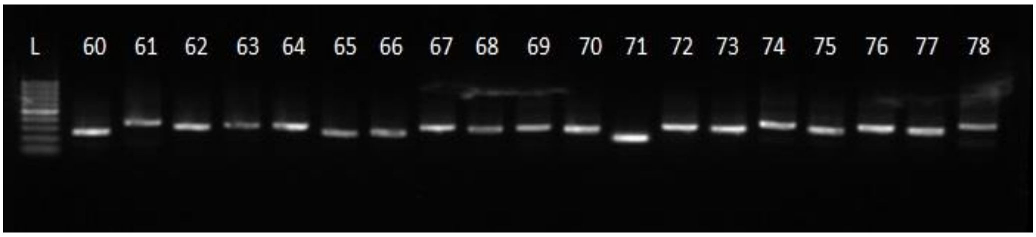

Figure 13: Lane L, 100-bp DNA ladder; lanes 60-78: the variable PCR product of spa.

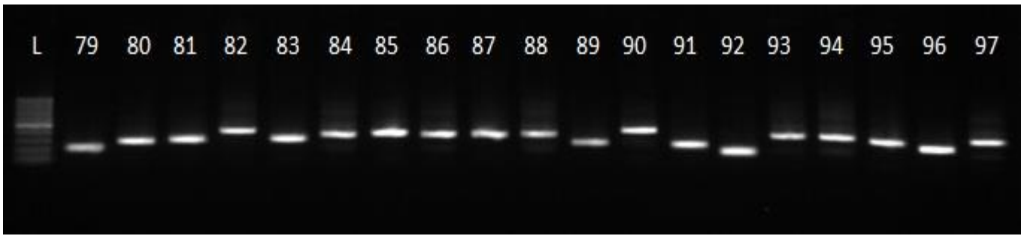

Figure 14: Lane L, 100-bp DNA ladder; lanes 79-97: the variable PCR product of spa.

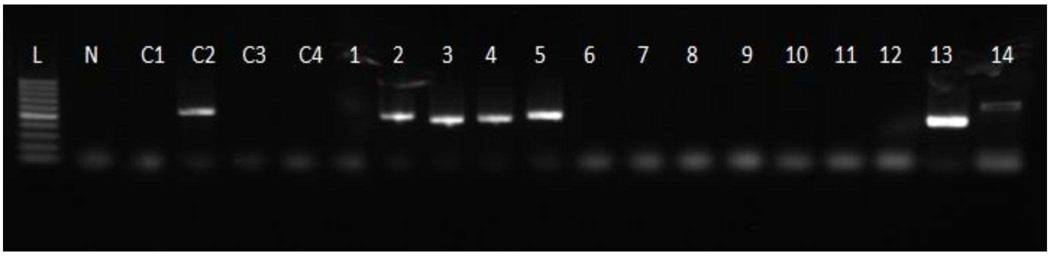

Figure 15: Lane L, 100-bp DNA ladder; lanes N, C1, C3 and C4: negative control; lane C2: PVL positive control amplicon (433-bp); lanes 1,6,7,8,9,10,11,12 and 4: no PCR product; lanes 2-5 and 13 PVL amplicon (433-bp).

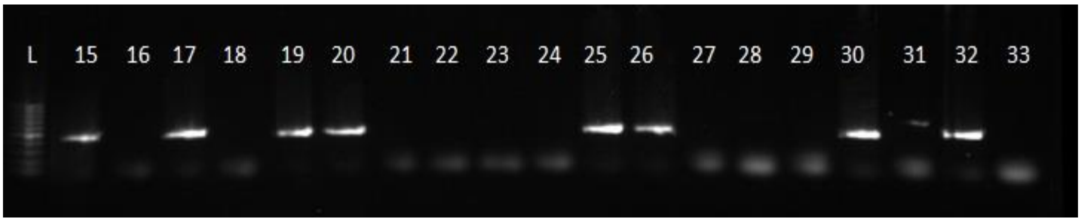

Figure 16. Lane L, 100-bpDNAladder; lanes 5,17,19-20,25-26,30 and 32 PVL amplicon (433-bp); lanes 16,18,21-24,27-29,31 and 33 : no PCR product

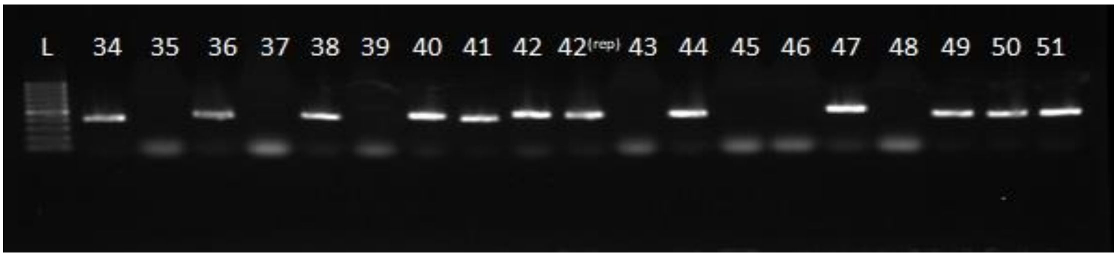

Figure 17: Lane L, 100-bpDNAladder; lanes 34,36,38,40-42,44,47 and 49-51 PVL amplicon (433-bp); lanes 35,37,39,43,45-46 and 48: no PCR product

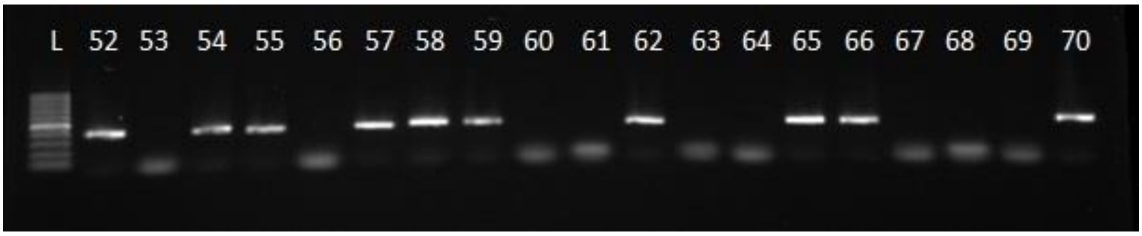

Figure 18: Lane L, 100-bpDNAladder; lanes 52, 54-55,57-59,62,65-66 and 70 PVL amplicon (433-bp); lanes 53,56,60-61,63-64 and 67-69: no PCR product

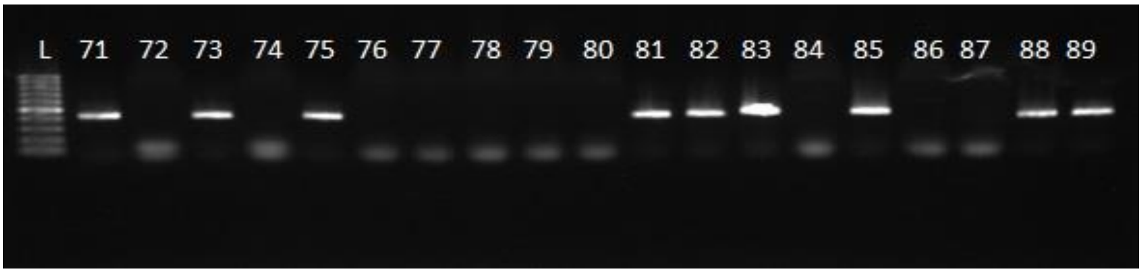

Figure 19: Lane L, 100-bpDNAladder; lanes 71,73,75,81-83,85 and 88-89 PVL amplicon (433-bp); lanes 72,74,76-80,84 and 86-87: no PCR product

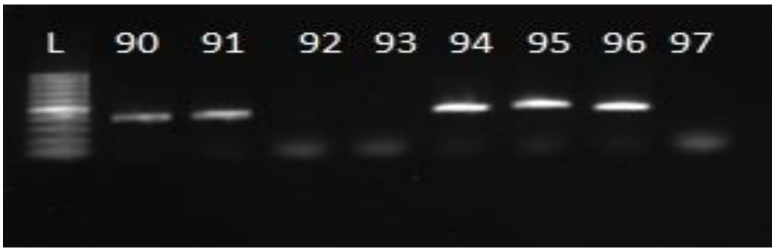

Figure 20. Lane L, 100-bp DNA ladder; lanes 90-91 and 94-96 PVL amplicon (433-bp); lanes 92-93: no PCR product
